# New report of two Cerambycinae tribes in South Korea: Stenopterini and Thraniini (Coleoptera, Cerambycidae)

**DOI:** 10.3897/BDJ.10.e81832

**Published:** 2022-05-12

**Authors:** Seunghyun Lee, Seunghwan Oh, Jinbae Seung, Hyunkyu Jang, Minhyeuk Lee, Woong Choi, Seunghwan Lee

**Affiliations:** 1 Key Laboratory of Zoological Systematics and Evolution, Institute of Zoology, Chinese Academy of Sciences, Beijing, Republic of Korea Key Laboratory of Zoological Systematics and Evolution, Institute of Zoology, Chinese Academy of Sciences Beijing Republic of Korea; 2 Manseung Blgd., 49, Myeongseong-ro 149-gil, Galmal-eup, Cheorwon, Republic of Korea Manseung Blgd., 49, Myeongseong-ro 149-gil, Galmal-eup Cheorwon Republic of Korea; 3 Insect Biosystematics Laboratory, Department of Agricultural Biotechnology, Seoul National University, Seoul, Republic of Korea Insect Biosystematics Laboratory, Department of Agricultural Biotechnology, Seoul National University Seoul Republic of Korea; 4 1810-1502, 69, Sangil-ro, Bucheon, Republic of Korea 1810-1502, 69, Sangil-ro Bucheon Republic of Korea; 5 Korea National Park Service, Wonju, Republic of Korea Korea National Park Service Wonju Republic of Korea; 6 305-403, Sechangnamsunhwan-ro, Namdong-gu, Incheon, Republic of Korea 305-403, Sechangnamsunhwan-ro, Namdong-gu Incheon Republic of Korea; 7 Research Institute for Agricultural and Life Sciences, Seoul National University, Seoul, Republic of Korea Research Institute for Agricultural and Life Sciences, Seoul National University Seoul Republic of Korea

## Abstract

**Background:**

Despite the recent advancement of faunal research of longhorned beetles in South Korea, the number of tribes of Cerambycinae has remained at 16 during the last 40 years.

**New information:**

In this paper, two cerambycine tribes, Stenopterini Gistel, 1848 and Thraniini Gahan, 1906, are reported for the first time in Korea by species Merionoeda (Macromolorchus) hirsuta (Mitono & Nishimura, 1936) and *Thraniusvariegatus* Bates, 1873, respectively. Morphological comments, biological observations and illustrations of both species are provided. An updated key to tribes of Korean cerambycinae is also provided.

## Introduction

The subfamily Cerambycinae (Coleoptera, Cerambycidae) is the second-largest group amongst six cerambycid subfamilies (sensu Nie et al. 2020: Cerambycinae, Dorcasominae, Lamiinae, Lepturinae, Spondylidinae, Prioninae), comprising approximately 1,700 genera and 11,000 species globally (Švácha and Lawrence 2014, Tavakilian and Chevillotte 2021). Cerambycinae is also the second-largest subfamily as well in the Korean fauna and is represented by 119 species from 16 tribes (Jang et al. 2015, Oh and Jang 2015, Niisato and Oh 2016, Lee and Lee 2016, Lee and Lee 2018, Lee et al. 2021). Even though numerous papers on their diversity (Han and Lyu 2010, Lim et al. 2012, Lim et al. 2013, Oh 2013, Lee et al. 2015, Oh and Jang 2015, Lee and Lee 2016, Niisato and Oh 2016, Lee and Lee 2018, Lee et al. 2021) and a few faunal reviews (Lee 1987, Lee 1982, [Bibr B7666478][Bibr B7666532], Lee 1982, Jang et al. 2015), the number of cerambycine tribes has remained at 16 (sometimes 15, depending on the tribal classification, see Table 1) during the last 40 years. In this study, we add the tribes Stenopterini Gistel, 1848 and Thraniini Gahan, 1906 to the Korean fauna by species Merionoeda (Macromolorchus) hirsuta (Mitono & Nishimura, 1936) and *Thraniusvariegatus* Bates, 1873, respectively.

## Materials and methods

Samples used in this study were deposited in SNU (Seoul National University) and private collections of H. Jang and S. Oh. Photographs of dorsal and ventral habitus were captured by a Canon digital camera EOS 80d, Canon MP-E 65 mm f/2.8 1–5× macro lens or Tamron SP 60 mm F/2.0 lens mounted. Multiple image stacking was conducted by Zerene Stacker 1.04 software (Zerene Systems 2014; http://www.zerenesystems.com/cms/stacker). To examine male and female genitalia, the specimens were relaxed in distilled water for two to four hours at room temperature. Then the genitalia were separated from the last abdomen segment using a hooked pin or forceps, without removing the abdomen. Separated genitals were immersed in 10% potassium hydroxide (KOH) solution at room temperature for eight to twelve hours, depending on the sample condition. For the illustration of genital structure, a microscope (DM 4000B, Leica Microsystem, Wetzlar, Germany) with a USB digital camera (Infinity3, Lumenera Corporation, Ottawa, Ontario) was used.

## Taxon treatments

### Merionoeda (Macromolorchus) hirsuta

(Mitono & Nishimura, 1936)

3EE3703B-C3CA-5C6F-A976-B70730B6D173

Hakata hirsuta Mitono & Nishimura, 1936: 34.
Hakata klapperichi Tippmann, 1955: 100.

#### Materials

- **Type status:** Other material. **Occurrence:** recordedBy: S.H. Oh; individualCount: 11; sex: 9♂, 2♀; lifeStage: adult; **Taxon:** scientificName: Merionoeda (Macromolorchus) hirsuta (Mitono & Nishimura, 1936); **Location:** country: South Korea; stateProvince: Jeollanam-do; locality: Geumja-ri, Busan-myeon, Jangheung-gun; **Event:** eventDate: 17.vii.2019; **Record Level:** institutionCode: Private Collection of S. H. Oh
- **Type status:** Other material. **Occurrence:** recordedBy: H. Jang; individualCount: 139; sex: 137♂, 2♀; lifeStage: adult; **Taxon:** scientificName: Merionoeda (Macromolorchus) hirsuta (Mitono & Nishimura, 1936); **Location:** country: South Korea; stateProvince: Jeollanam-do; locality: Geumja-ri, Busan-myeon, Jangheung-gun; **Event:** eventDate: 17.vii.2021; **Record Level:** institutionCode: Private collection of H. Jang

#### Description

**Figure 1.**
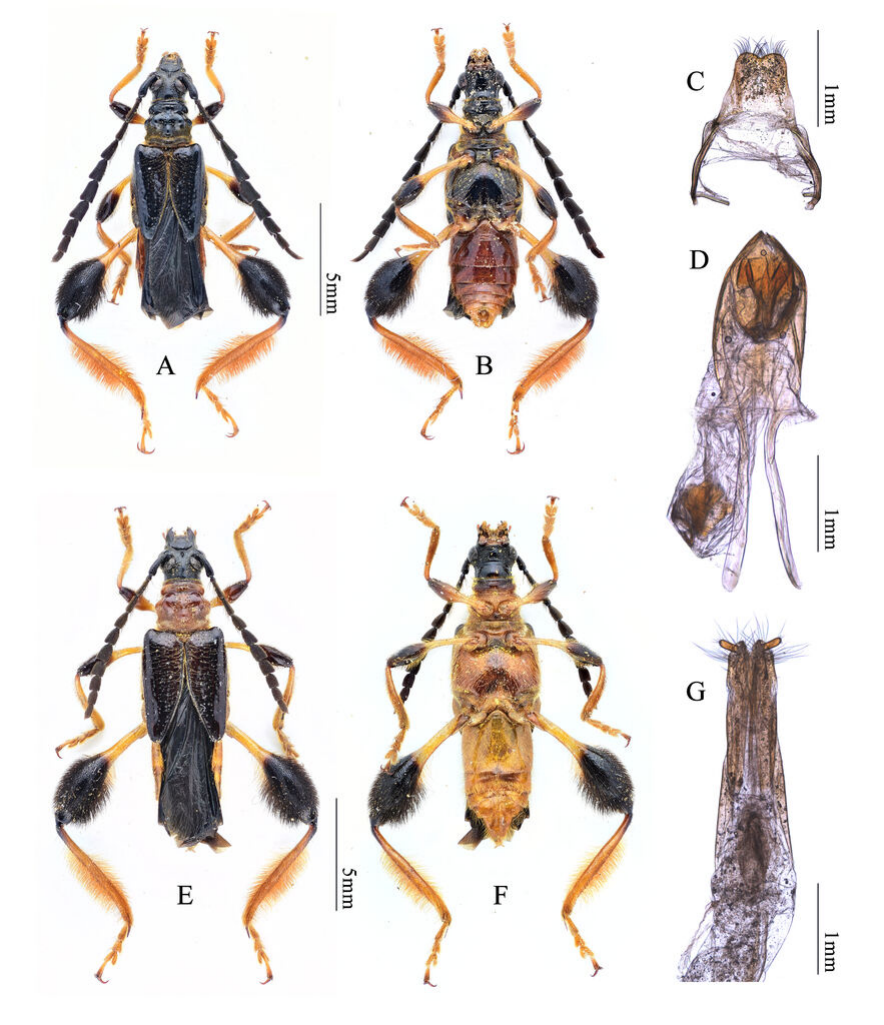
Merionoeda (Macromolorchus) hirsuta (Mitono & Nishimura, 1936). **A, B** Male habitus; **C** Tegmen; **D** Penis; **E, F** Female habitus; **G** Ovipositor.

Body length 10-14 mm (Fig. 1A and B). Head black, minutely and densely punctuated with sparse golden setae, frons with short, but distinct longitudinal median suture. Antennae black, slightly shorter than body length in male, slightly longer than half of body length in female. Pronotum black in male, orange in female, with two longitudinal row of punctuations present medially, moderately pubescent. Prosternum black in male, orange in female, anteriorly with shallow transverse groove and with pale setae, prosternal process well-developed with apex widened. Abdomen orange in female, dark brown with brighter posterior segments in male, sparsely pubescent. Scutellum almost semicircular, black in male, orange in female. Elytra metallic black, almost half as long as body length, narrowed posterolaterally with somewhat rounded and unarmed apex. Legs orange with distal half of femora black, moderately pubescent on fore- and mid-legs, densely pubescent with long setae on hind legs, hind femora distinctly swollen. Tegmen apically blunt with short incision on middle, parameres indistinct, apex with long and dense setae (Fig. 1C). Penis bullet-like with sharp apex, almost as long as dorsal struts (Fig. 1D). Ovipositor gradually and slightly narrowed towards apex, slightly bilobed at apex, apically hairy with short styli (Fig. 1G).

#### Distribution

Korea (new record), China, Japan, Taiwan.

#### Notes

Emergence begins in early July in the southern part of the Korean Peninsula. Beetles are most active in warm clear weather and visit the male flower of *Mallotusjaponicus* (Thunb.) Muell. Arg. The population size in the site seems remarkably high as 139 beetles were caught in a few hours. The number of males visiting flowers is approximately ten times larger than that of females.

### Thranius variegatus

Bates, 1873

0A4050CF-1B1A-5FB7-8455-56C51BBE6FB9

Thranius variegatus Bates, 1873: 196.
Thranius sapporensis Kano, 1933: 132.

#### Materials

- **Type status:** Other material. **Occurrence:** recordedBy: Seunghyun Lee; individualCount: 1; sex: 1♀; lifeStage: adult; **Taxon:** scientificName: *Thraniusvariegatus* Bates, 1873; **Location:** country: South Korea; stateProvince: Jeju-do; locality: Namjo-ro, Jocheon-eup, Jeju-si; **Event:** eventDate: 28.vii.2016; **Record Level:** institutionCode: SNU

#### Description

**Figure 2.**
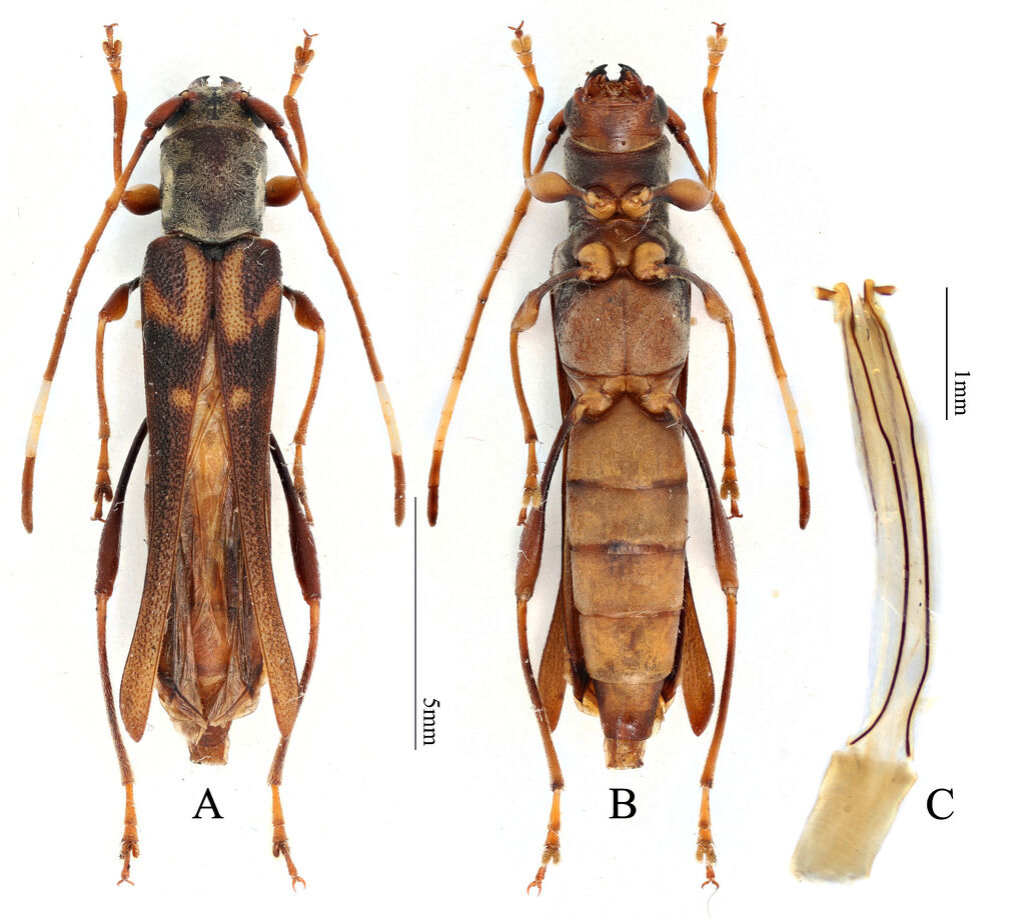
*Thraniusvariegatus* Bates, 1873. **A, B** Female habitus; **C** Ovipositor.

Body length of examined female 13.4 mm (Fig. 2A and B). Head dark brown, densely covered with pale decumbent setae, frons with longitudinal median suture. Antennae brown, except for antennomeres VIII-IX white, notably shorter than body length in female. Pronotum dark brown, densely covered with pale decumbent setae. Ventrum light brown, densely pubescent with minute decumbent setae. Scutellum black, almost semicircular. Elytra dark brown, with L-shaped brighter marking near humeri, narrowed posteriorly, partly exposing posteromedial region of abdomen. Legs brown to dark brown, moderately pubescent with decumbent setae, hind femora slightly swollen. Ovipositor approximately 7.5× longer than wide, almost parallel on side, distinctly, but shortly bilobed at apex and with short apical styli (Fig. 2C).

#### Distribution

Korea (new record), Japan, Taiwan.

#### Notes

No additional beetles have been collected after the first discovery, though we launched numerous flight intercept traps every year at the same spot.

## Identification Keys

### Updated key to tribes of the subfamily Cerambycinae in Korea (modified from Lee, 1987)

**Table:** 

1	Abdomen not fully covered by elytra, abdominal segments partly exposed	2
–	Abdomen completely covered by elytra	4
2	Elytra posteriorly narrowed, abdomen posteromedially and posterolaterally exposed	**Thraniini**
–	Elytra posteriorly shortened, abdomen posteriorly exposed	3
3	Elytal apex weakly angulated, hind legs with dense setae, female 4th sternite posteriorly with dense setae	**Stenopterini**
–	Elytral apex rounded, hind leg sparsely pubescent, female sternite plain	**Molorchini**
4	Antennomeres V-X flat, prominently serrate	**Pyrestini**
–	All antennomeres uniformly filiform	5
5	Prothorax distinctly longer than wide	6
–	Prothorax not longer than wide	9
6	Prothorax slightly curved laterally, without lateral tubercles	7
–	Prothorax parallel-sided with strong lateral tubercles	8
7	Antennomeres III-XI with spine at inner apex	**Phoracanthini**
–	Each antennomere without spine at inner apex	**Callidiopini**
8	Hind femur thickened rapidly on apical half	**Obriini**
–	Hind femur gradually thickened towards apex	**Stenhomalini**
9	Compound eyes coarsely faceted	10
–	Compound eyes finely faceted	12
10	Prosternal process expanded, genae moderately wide	11
–	Prosternal process not expanded, genae distinctly short	**Hesperophanini**
11	Pronotum with irregular transverse or longitudinal grooves on disc	**Cerambycini**
–	Pronotum with regular punctation on disc	**Xystrocerini**
12	Body more or less flat, elytra completely flat, except lateral margin in lateral view	13
–	Body almost cylindrical, elytra slightly convex in lateral view	17
13	Metaventrite expanded towards mesocoxal cavity, mesocoxal cavity closed	**Cleomenini**
–	Metaventrite not expanded towards mesocoxal cavity, mesocoxal cavity opened	14
14	Antennomere III-IX with black setae on apical half; elytra sky-blue with black markings	**Compsocerini**
–	Antennomere III-IX without black setae; elytra not sky-blue	15
15	Prothorax laterally without tubercle	**Callidiini**
–	Prothorax laterally with spine-like tubercle	16
16	Hind tibia flat, elytra green or metallic navy with two yellow bands	**Callichromatini**
–	Hind tibia cylindrical, elytra black and red	
17	Metepimeron expanded towards abdominal sternite	**Clytini**
–	Metepimeron not expanded towards abdominal sternite	**Anaglyptini**

## Supplementary Material

XML Treatment for Merionoeda (Macromolorchus) hirsuta

XML Treatment for Thranius variegatus

## Figures and Tables

**Table 1.** Tribal classification of Korean Cerambycinae by various authors (bold words indicate tribal names different from the current classification; * = tribe new to Korean fauna).

**Lee 1982**	**Lee 1987**	**Jang et al. 2015**	**This study**
Anaglyptini	Anaglyptini	Anaglyptini	Anaglyptini
Callichromatini	Callichromatini	Callichromatini	Callichromatini
Callidiini	Callidiini	Callidiini	Callidiini
Callidiopini	Callidiopini	Callidiopini	Callidiopini
Cerambycini	Cerambycini	Cerambycini	Cerambycini
Cleomenini	Cleomenini	Cleomenini	Cleomenini
Clytini	Clytini	Clytini	Clytini
Hesperophanini	Hesperophanini	Hesperophanini	Hesperophanini
Molorchini	Molorchini	Molorchini	Molorchini
**Obriini** (= **Obriini + Stenhomalini)**	**Obriini (= Obriini + Stenhomalini)**	Obriini	Obriini
		Stenhomalini	Stenhomalini
Phoracanthini	Phoracanthini	Phoracanthini	Phoracanthini
Purpuricenini	Purpuricenini	Purpuricenini	Trachyderini
Pyrestini	Pyrestini	Pyrestni	Pyrestini
Rosaliini	Rosaliini	Rosaliini	Compsocerini
**Methiini (=Xystrocerini)**	**Methiini (=Xystrocerini)**	Xystrocerini	Xystrocerini
			Stenopterini*
			Thraniini*
